# Facilitating CAD/CAM nasoalveolar molding therapy with a novel click-in system for nasal stents ensuring a quick and user-friendly chairside nasal stent exchange

**DOI:** 10.1038/s41598-018-29960-z

**Published:** 2018-08-14

**Authors:** Florian D. Grill, Lucas M. Ritschl, Hannes Dikel, Andrea Rau, Maximilian Roth, Markus Eblenkamp, Klaus-Dietrich Wolff, Denys J. Loeffelbein, Franz X. Bauer

**Affiliations:** 1 Department of Oral and Maxillofacial Surgery, Technische Universität München, München, Germany; 2 Institute of Medical and Polymer Engineering, Technische Universität München, München, Germany; 3 Department of Oral and Maxillofacial Surgery, Friedrich Alexander Universität Erlangen-Nürnberg, Erlangen, Germany; 4 Department of Oral and Maxillofacial Surgery, Helios Hospital Munich West, Teaching Hospital of Ludwig-Maximilians-Universiität, München, Germany

## Abstract

Nasoalveolar molding (NAM) aims to improve nasal symmetry with a nasal stent in cleft lip and palate (CLP) patients. When plates have to be exchanged because of dentoalveolar growth or cleft reduction, the nasal stent has to be mounted onto a new plate. This procedure elongates visiting hours for patients and parents or requires second treatment sessions. This study introduces a quick-lock additive manufacturing solution for chairside nasal stent exchange called RapidNAM. A novel taping retention pin has been designed that enables nasal stent insertion. Patients with unilateral CLP were included in this study. Plaster models were digitalized and measured by two independent observers. Two methods of CAD/CAM-molding therapies were compared: (i) conventional adhesion of a nasal stent (CAD/CAM NAM); (ii) quick-lock system in which the nasal stent was transferred to another plate (RapidNAM). CAD/CAM NAM and its refinement RapidNAM significantly increased the cleft-side nasal height and tilted the nose towards symmetry. The quick-lock system minimizes wire adaptations, since the pre-existing stent can be reused. The new nasal stent development seems a feasible solution to minimize visiting hours but with clinically satisfactory results. This new nasal stent system combines traditional elements of NAM with CAD/CAM-technology.

## Introduction

Nasoalveolar molding (NAM) is a presurgical treatment modality of cleft lip and palates (CLP). It is a combination of an approximation and alignment of the alveolar segments in the first treatment period and increasing nasal symmetry. Latter usually starts in the second half of the treatment period when the columella is lengthened and the cleft-side nostril is formed^1^. On the cleft side, the alar base is situated laterally, resulting in a sigmoid shape of the nostril. This displacement is the target of the nasal molding part of NAM, taking advantage of the ability to mold cartilage within the first few weeks of life^2,3^. NAM benefits the surgical outcome by reducing tension on the tissues with long-term results^1,3^. Furthermore, the number of nasal revisions can be reduced^4^. Usually, this is carried out by regular activation of the nasal stent integrated into the NAM plate and regular activation^5^. However, various approaches are available concerning the design^6^. The most commonly known is the regular steel wire, for instance, in the Grayson or Figueroa technique^7,8^. However, a construction that is fixed at the forehead and that applies dragging instead of pushing forces has also been tried, called the nasal alar elevator. Results show an improvement of the columella angle and nostril symmetry^9^. The steel wire itself has also experienced modifications by means of different materials and wire designs. For example, Nagraj and colleagues integrated double loops in their titanium molybdenum alloy wire. This decreased not only the treatment sessions, but also the number of follow-up sessions^10^. Nevertheless, when the plate has to be exchanged because of variations in the dimensions of the maxilla, the nasal stent has to be newly integrated after checking the fit and the attachment of the plate on the alveolar arches. This involves either a second treatment appointment, or the treatment session has to be disrupted by stent integration by the dental technician. This may contribute to the concerns that NAM is a time-consuming treatment procedure^11,12^. Therefore, as part of the semi-automation of CAD/CAM assisted NAM, we have developed RapidNAM, which intends to refine the already used CAD/CAM-technology in NAM therapy by using the feasibility of virtual modelling and advantages in preciseness of additive manufacturing. Our intention was to modify our pre-existing CAD/CAM solution for a virtual production of intraoral molding plates. This design also allows a quick chairside change of nasal stents thus reducing the duration of the treatment session, saving valuable time for the patients and their parents and for the practitioners. The presented system combines traditional and well-established features of NAM with the advantages of additive manufacturing.

## Patients, Materials and Methods

### Informed consent

All interactions with each patient in this study were performed with parental informed consent.

### Treatment Methods

In total, 14 healthy newborns requiring treatment of unilateral CLP were included in the study. In the analysis, two groups were formed: one group had been previously treated with conventionally CAD/CAM-intraoral molding plates that were designed digitally^13,14^, whereas the other group had been treated with RapidNAM, which included the novel nasal stent system. In both groups, impression-taking sessions from the nose and upper lip had been performed within the first few days of life and at the end of intraoral and nasal molding therapy when primary lip closure was carried out at the age of approximately 3–4 months^1^. The casts were digitalized with a 3D triangulation scanner with a resolution of 20 µm (3Shape D500, 3Shape; Copenhagen, Denmark)^13,15^. Both techniques involved the use of a stainless steel wire construction and a nasal bulb made of resin pattern. For nasal stent activation, the treatment groups attended weekly clinical controls. For extraoral tapings, the Grayson technique was used^7^.

### CAD/CAM-NAM

The CAD/CAM NAM group underwent the conventional method of stent retention. The removal of pin from the former plate was performed under less than one minute. To create sufficient retention, a notch was ground into the plate, the steel wire was attached, and then acrylic resin (Orthorcyl®, Dentaurum, Germany) was added and hardened. Creating the notch by grinding was done within one minute, however, the hardening of the used conventional resin pattern takes up to 20 minutes according to the manufacturer’s instructions. At our treatment center this takes usually two treatment sessions on the same day, the first involving a clinical check-up and marking the position of the future nasal pin on the plate. As an aid for the dental technician, a small wax rod was attached to the plate which indicates the desired length and bending of the future nasal stent. In the second treatment session, the molding plate that was remodeled and equipped with a new nasal stent by a dental technician, was inserted. A quick adjustment to the clinical situation is necessary and can usually be performed in one or two minutes. The technique corresponds to the traditional way of stent attachment with the related time consumption^1,16^.

### RapidNAM and Semi-Automated Plate Generation

**Figure 1:**
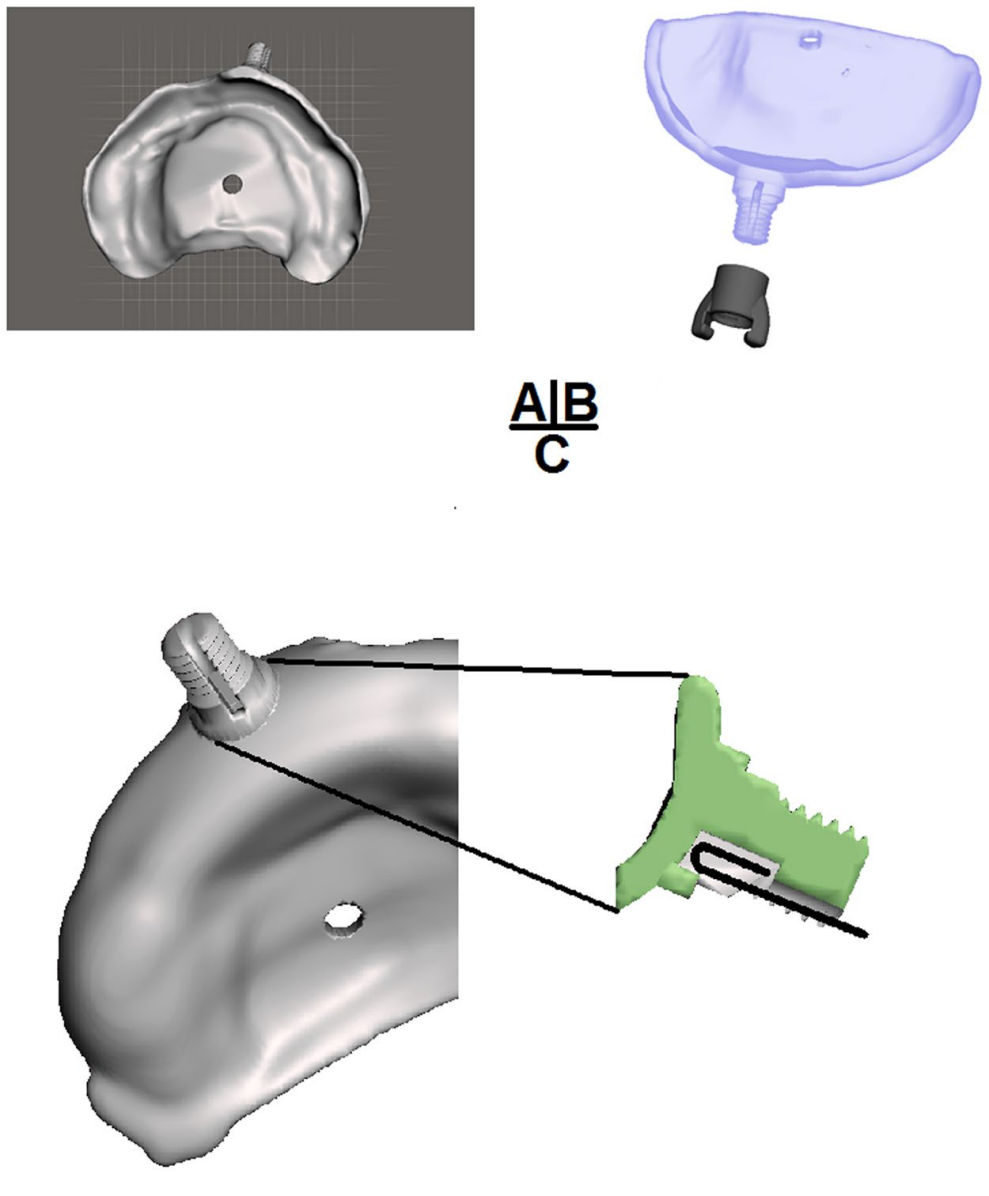
(**A**) Virtual RapidNAM plate with retention pin and screw thread. (**B**) Corresponding nut with retentions for buccal tapings. (**C**) Caudal view of the retention pin with the retention groove and profile view. The wire (black) can be clipped into the retentive slot.

The second group was treated with RapidNAM, based on a semi-automated production of intraoral molding plates^17^. For further facilitation, a novel retention system was developed. Four objectives had to be fulfilled: (i) compactness, (ii) stability, (iii) user-friendliness, and (iv) functionality. After multiple testing phases with various design approaches, a click-in system with a modified locking screw was chosen. An additional retention on the nut, which was laterally reinforced, allowed buccal taping to be attached. The taping with elastics at its end was attached to the retention arms on the tip of the nut. The steel wire needed a 4-mm-long 180°-knee and was inserted from a caudal direction into the retention pin. The guiding groove accepted a steel wire with a diameter of 0.9 mm. The screw thread had a definite orientation. This guaranteed that, after attachment of the nut, the orientation of the retentions for taping on the tip were always correct. The nut also pressed and locked the wire into the construction. The 180°-knee prevented sideway-shifting, and the nut ensured horizontal stability (Fig. 1). The insertion of a nasal stent or the change of the already used and adjusted pin to a new plate is possible in less than one minute and can be performed immediately. No extra time for the hardening of resin pattern is required whenever a new plate is necessary. After insertion of the stent to a new plate, the retention pin needs no further adjustments to touch the nostril at the correct site due to the invariable orientation of the guiding groove.

### Measuring Methods

**Figure 2:**
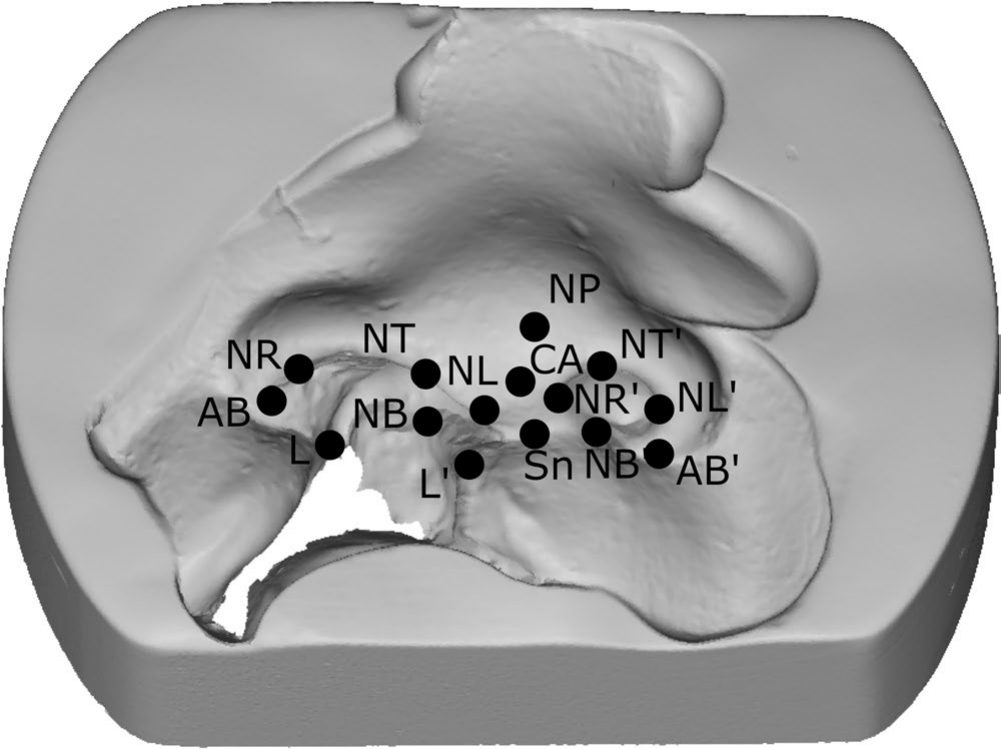
Selected landmarks.

The digitalized plaster models were virtually measured by using Python® (PYthon Software Foundation, version 2.7, Netherlands; The Scientific PYthon Development EnviRonment, Spyder Developer Community, version 2.3.4) based on selected landmarks as used in previous publications^1,13^. The following points on the nose were selected by two independent observers (Fig. 2): alar bases (AB, AB’), nasal tip (NT), subnasal point (SN), most anterior point of the columella (CA), top (NT, NT’) and bottom (NB, NB’) of the nostril on the healthy and the cleft side, inner rim to the left and right of each nostril (NL, NL’ and NR, NR’), and points that had the shortest connection line passing over the cleft on the vermilion of the lip (L, L’). The columella angle was defined as the angle formed by the lines crossing through [AB;AB’] and [Sn;CA].

For a standardized comparison of the previously known CAD/CAM-technique and RapidNAM, plaster models of CAD/CAM-NAM-treated children were re-measured digitally by using the same reproducible landmarks in both treatment groups.

### Statistical Analysis

Statistical analysis was carried out by using the R statistical environment with the user interface R Studio. For calculating differences between initial and final impression-taking sessions the Wilcoxon-signed rank test and the Wilcoxon exact test was used. A p-value of <0.05 was considered statistically significant. The plots show box plots with their upper and lower borders indicating the 1^st^ and 3^rd^ quartiles. The median is represented by the bar within the box^18,19^.

### Data availability

All data used for analysis in this study will be made available after publication.

## Results

In this pilot study, seven newborns with CLP were included who had been treated previously with CAD/CAM-NAM. In the RapidNAM-cohort, seven newborns with CLP were treated with the novel nasal stent system. For comparison purposes, the impressions of the CAD/CAM NAM cohort were re-measured digitally. One patient dropped out of NAM treatment because of parental difficulties in applying daily tapings, so that a regular drinking-plate was used instead.

### Attachment of retention pins on CAD/CAM and RapidNAM plates

#### Conventional CAD/CAM-NAM

The retention pin in CAD/CAM-NAM plates had to be inserted by the user by a Boolean operation. The correct inclination had to be determined manually by the user (Geomagic®, Studio 12, Morrisville, NC, USA). The attachment was a time-consuming procedure, since the position, angle, and plate thickness had also to be considered during the virtual design process^14,16^. In the printed plate, the nasal stent was inserted conventionally^8^. The retention of the nasal stent takes more time mostly due to technical requirements (hardening of resin pattern) but also due to the necessity of readjusting the nasal stent.

#### RapidNAM

**Figure 3:**
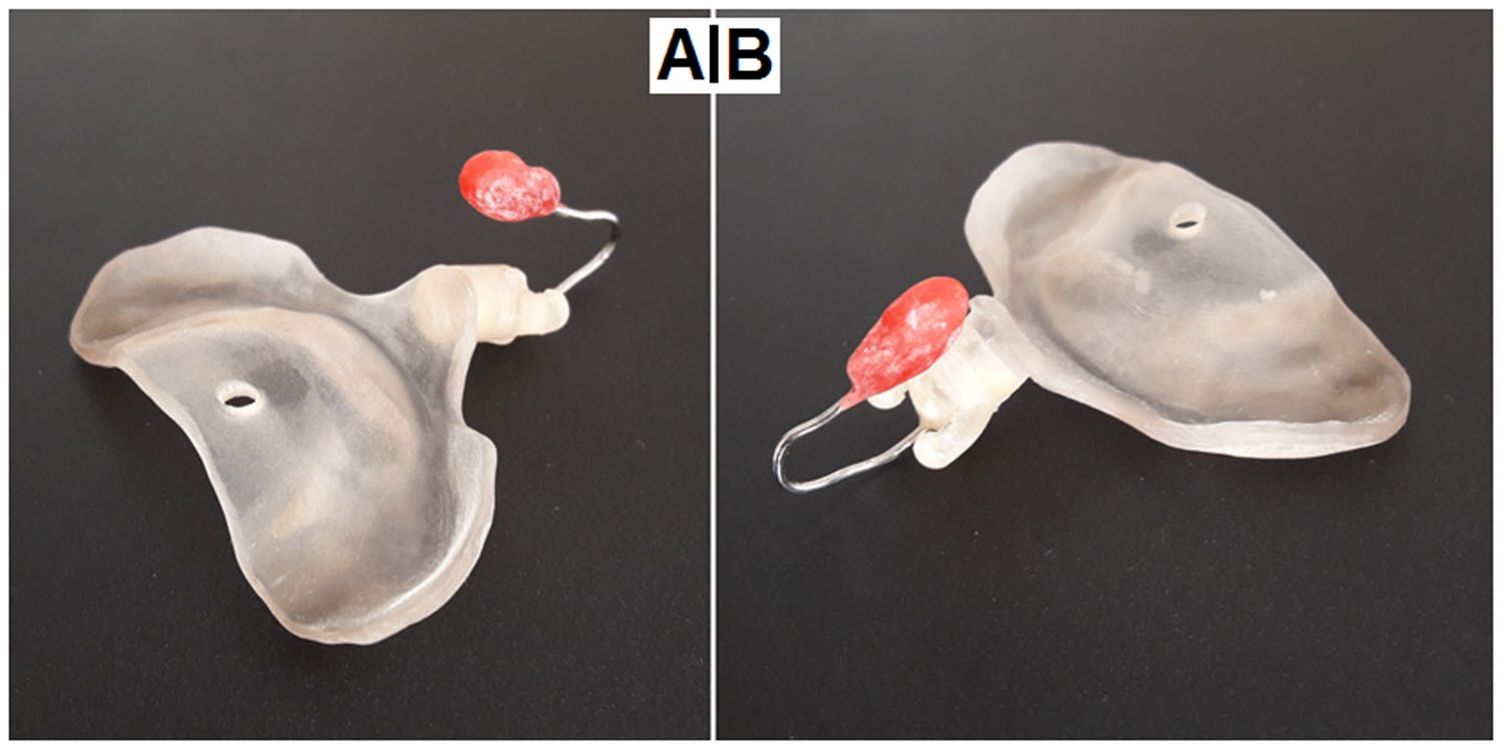
(**A**) Dorsal view of a RapidNAM plate with attached nasal stent. (**B**) Frontal view of a RapidNAM plate with attached nasal stent.

The RapidNAM-software application automatically inserts the retention pin at a recommended tilting angle of approximately 40° derived from traditional NAM^7,20^. The actual position is determined by choosing the desired position in the RapidNAM-graphical user interface (GUI) and requires no further manual interactions since the required groove for nasal stent retention is already included in the design. However, the cleft lip position and its soft-tissue situation have to be considered for space requirements. The plate design can occur even during the treatment session because of the GUI. When designed at a later time, a 3D photograph is helpful for finding the proper position. In one case, the pin position had to be corrected and moved further to the midline. In this case, the pin was manually removed, and acrylic resin was added and hardened. Depending on the buccal taping angle, the retention arms on the nut had to be lifted, and the retention therefore deepened. After two weeks of use, the retention within the pin had to be reinforced (because of wear) by a thin layer of acrylic resin. The stent was re-fixed by the screw and was stable again in all directions. Figure 3 shows an actual RapidNAM plate with attached nasal stent.

### Nasal Development

#### Conventional CAD/CAM-NAM

**Figure 4:**
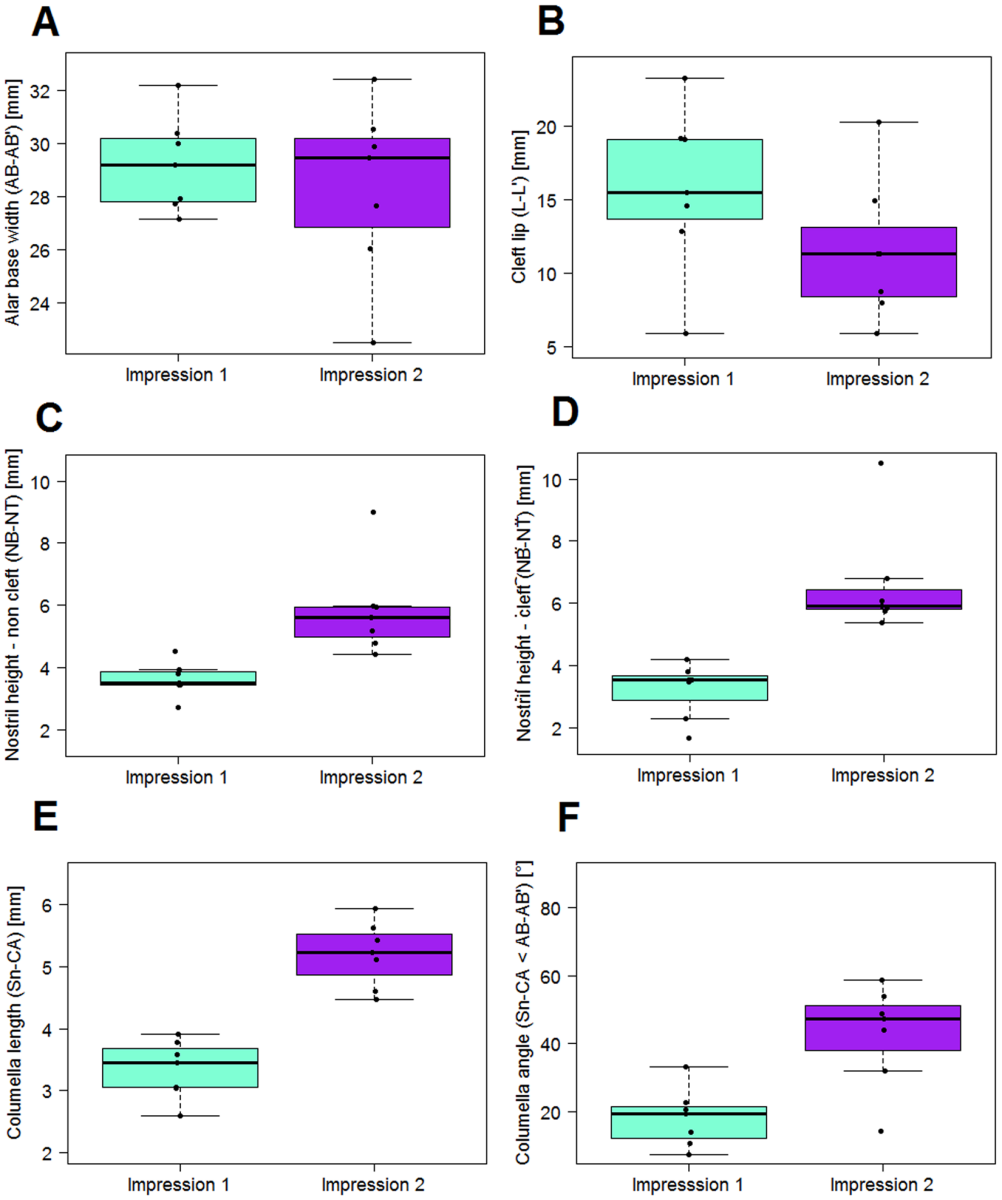
Statistical results before and after CAD/CAM-NAM treatment. (**A**) Transversal dimensions between alar bases. (**B**) Transversal dimensions of cleft lip. (**C**) Nasal height on non-cleft side. (**D**) Nasal height on cleft side. (**E**) Columella length. (**F**) Nasolabial angle.

The transversal nasal dimensions are captured by the distance between the alar bases (AB and AB’) (Fig. 4, Table 1). The dimension decreased by 0.9 mm starting with a mean of 29.2 mm. The cleft lip was also narrowed by 4.3 mm. Initial cleft size was 15.8 mm after birth and 11.5 mm after NAM treatment. The cleft-affected nostril was increased by 3.4 mm from a mean of 3.2 to 6.6 mm. The non-affected nostril experienced no explicit treatment. Here, the increase was 2.2 mm in the same time period and was statistically significant (Table 2). The nostril width on the cleft side was initially 17.4 mm and was reduced by 2.2 mm. The non-cleft side experienced a reduction of 0.4 mm. The columella was lengthened by 1.8 mm, an increase of 55%. The columella angle was increased from a mean of 18.3° to 42.7°. The ratio of nostril height cleft/non-cleft was increased from a mean of 0.881 in the initial impression (SD: 0.211, median: 0.902) to a mean of 1.136 (SD: 0.121, median: 1.119) at the end of NAM-treatment. The difference was found to be statistically significant with a p-value of 0.026

**Table 1:** Distances between reference points with differences before and after CAD/CAM-NAM therapy.

DISTANCES	MIN.	25%-QUARTILE	MEDIAN	MEAN	75%-QUARTILE	MAX.	SD
AB – AB′ IMPRESSION 1	27.2	27.8	29.2	29.2	30.2	32.2	1.8
AB – AB′ IMPRESSION 2	22.5	26.8	29.5	28.4	30.2	32.4	3.3
AB – AB′ DIFFERENCE	−6.7	−1.1	0.2	−0.9	0.5	1.6	2.8
L – L′ IMPRESSION 1	5.9	13.7	15.5	15.8	19.1	23.3	5.6
L – L′ IMPRESSION 2	5.9	8.4	11.3	11.5	13.1	20.2	4.8
L – L′ DIFFERENCE	−8.4	−7.3	−6.6	−4.3	−0.7	1.1	4.0
NB^(^′^)^ - NT^(^′^)^ NON-CLEFT IMPRESSION 1	2.7	3.4	3.5	3.6	3.9	4.5	0.6
NB^(^′^)^ - NT^(^′^)^ NON-CLEFT IMPRESSION 2	4.4	5.0	5.6	5.8	6.0	9.0	1.5
NB^(^′^)^ - NT^(^′^)^ NON-CLEFT DIFFERENCE	0.7	1.1	1.7	2.2	2.7	5.6	1.7
NB^(^′^)^ - NT^(^′^)^ CLEFT IMPRESSION 1	1.7	2.9	3.6	3.2	3.7	4.2	0.9
NB^(^′^)^ - NT^(^′^)^ CLEFT IMPRESSION 2	5.4	5.8	5.9	6.6	6.4	10.5	1.8
NB^(^′^)^ - NT^(^′^)^ CLEFT DIFFERENCE	1.8	2.1	2.5	3.4	4.5	6.3	1.7
SN - CA IMPRESSION 1	2.6	3.0	3.5	3.3	3.7	3.9	0.5
SN - CA IMPRESSION 2	4.5	4.9	5.2	5.2	5.5	5.9	0.5
SN – CA DIFFERENCE	1.1	1.5	1.8	1.8	2.0	2.9	0.6
SN-CA ∢ AB-AB IMPRESSION 1 [ANGLE]	7.4	12.4	19.4	18.3	21.7	33.2	8.6
SN-CA ∢ AB-AB IMPRESSION 2 [ANGLE]	14.4	37.9	47.3	42.7	51.3	58.7	15.0
SN-CA ∢ AB-AB DIFFERENCE [ANGLE]	−8.2	19.1	27.9	24.4	34.9	43.2	17.3
NR^(^′^)^ - NL^(^′^)^ NON-CLEFT IMPRESSION 1	5.8	7.7	8.1	7.9	8.3	9.3	1.1
NR^(^′^)^ - NL^(^′^)^ NON-CLEFT IMPRESSION 2	5.9	6.9	7.2	7.4	8.1	8.9	1.0
NR^(^′^)^ - NL^(^′^)^ NON-CLEFT DIFFERENCE	−2.0	−1.1	0.1	−0.4	0.2	0.8	1.0
NR^(^′^)^ - NL^(^′^)^ CLEFT IMPRESSION 1	15.4	16.6	16.9	17.4	17.6	20.8	1.7
NR^(^′^)^ - NL^(^′^)^ CLEFT IMPRESSION 2	11.6	14.8	15.0	15.2	16.3	17.5	2.0
NR^(^′^)^ - NL^(^′^)^ CLEFT DIFFERENCE	−5.1	−2.8	−2.2	−2.2	−1.0	−0.3	1.7

**Table 2:** P-values for differences before and after CAD/CAM and RapidNAM therapy.

CAD/CAM NAM		RapidNAM	
DISTANCES	P-VALUES	DISTANCES	P-VALUES
AB - AB′	0.805	AB - AB	0.792
NR^(^′^)^ - NL^(^′^)^ CLEFT	*0.053	NR - NL CLEFT	0.662
NR^(^′^)^ - NL^(^′^)^ NON-CLEFT	0.535	NR′ - NL′ NON-CLEFT	0.792
NB^(^′^)^ - NT^(^′^)^ CLEFT	*0.001	NB - NT′ CLEFT	*0.017
NB^(^′^)^ - NT^(^′^)^ NON-CLEFT	*0.001	NB′ - NT′ NON-CLEFT	0.08
SN - CA	*0.001	SN - CA	0.247
SN - NP	*0.007	SN - NP	0.429
L - L′	0.165	L - L′	0.177
SN-CA ∢ AB-AB′	*0.011	SN-CA ∢ AB-AB	0.247

*P-values > 0.05 were found to be statistically significant.

#### RapidNAM

**Figure 5:**
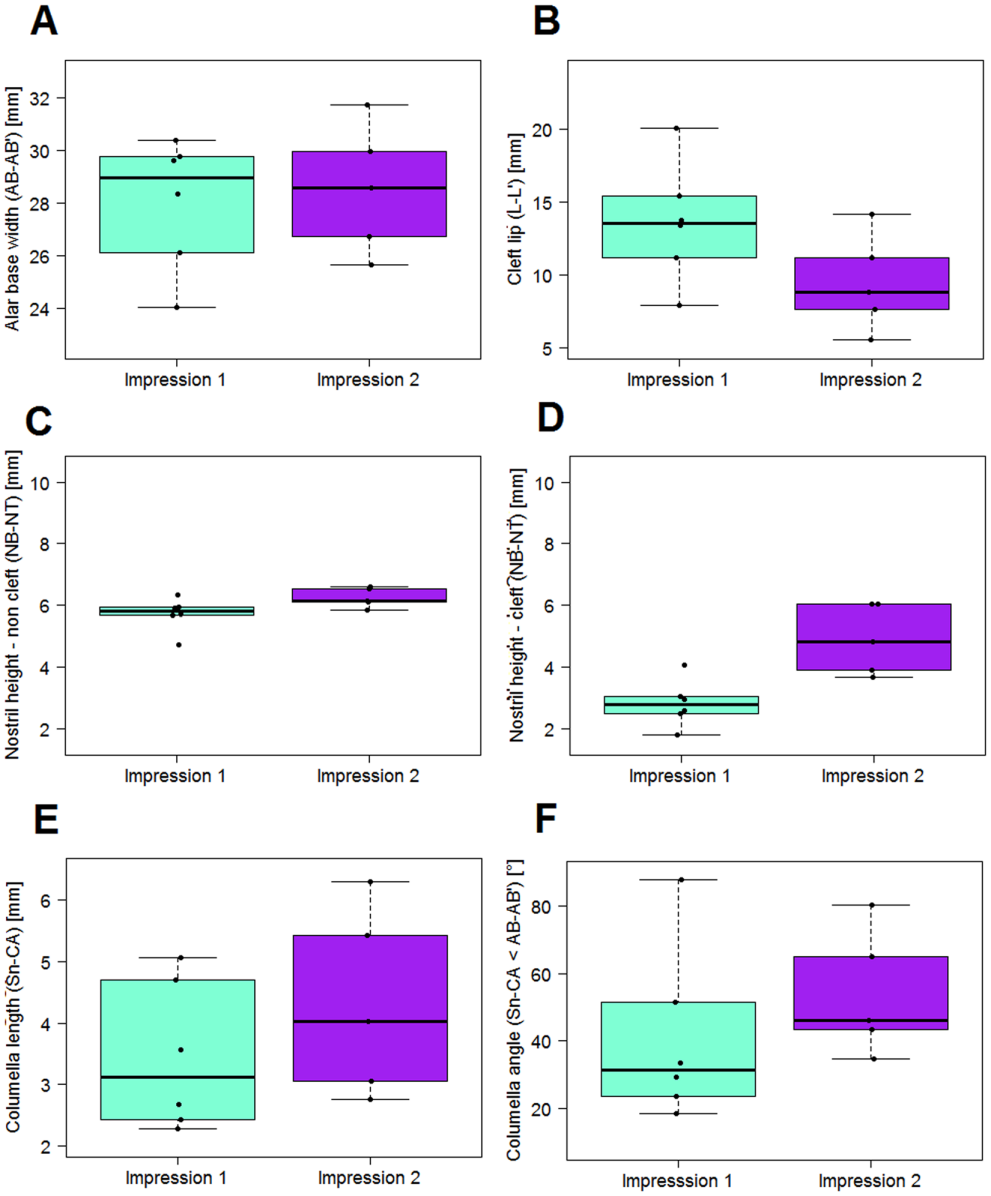
Statistical results before and after RapidNAM treatment. (**A**) Transversal dimensions between alar bases. (**B**) Transversal dimensions of cleft lip. (**C**) Nasal height on non-cleft side. (**D**) Nasal height on cleft side. (**E**) Columella length. (**F**) Nasolabial angle.

From 7 included patients, six patients were treated with RapidNAM. In one case, NAM had to be cancelled because of parental difficulties in handling daily taping. In this case, a regular drinking plate was used without a nasal pin. In another case, only the initial cast was included in the analysis because of severe artifacts in the second impression at various landmark positions. Therefore, the initial descriptive figures and tables show n = 6 for the initial impression and n = 5 for the second impression. The differences mentioned were correctly calculated with n = 5. The distance between the alar bases representing the nasal width was reduced by 1.0 starting with 29.0 mm (Fig. 5, Table 3). The two parts of the upper lip showed an initial cleft size of 13.6 mm and after treatment 9.5 mm, which is a mean reduction of 2.8 mm. The cleft-affected nostril was increased by 2.1 mm from a mean of 2.8 to 4.9 mm, a statistically significant difference (Table 2). The non-affected nostril experienced no treatment, and the increase was 0.6 mm in the same time period. The nostril width on the cleft side was initially 15.1 mm and was reduced by 0.6 mm. The non-cleft side experienced an increase of 0.3 mm. The columella was lengthened by 1.1 mm, an increase of 31%. The nasolabial angle was increased from a mean of 40.7° to 53.9°, which is an increase of 11.8°. The ratio of nostril height cleft/non-cleft was increased from a mean of 0.493 (SD: 0.113, median: 0.519) in the first impression to a mean of 0.785 (SD: 0.190, median 0.729) in the second impression. The difference was found to be statistically significant with a p-value of 0.017.

**Table 3:** Distances between reference points with differences before and after RapidNAM therapy.

DISTANCES	MIN.	1ST QUARTILE	MEDIAN	MEAN	3RD QUARTILE	MAX.	SD
AB – AB′ IMPRESSION 1	24.0	26.7	29.0	28.0	29.7	30.4	2.5
AB – AB′ IMPRESSION 2	25.7	26.7	28.6	28.5	30.0	31.7	2.4
AB – AB′ DIFFERENCE	0.2	0.2	0.6	1.0	1.6	2.1	0.9
L – L‘ IMPRESSION 1	8.0	11.7	13.6	13.6	15.0	20.1	4.1
L – L′ IMPRESSION 2	5.5	7.7	8.8	9.5	11.2	14.2	3.3
L – L′ DIFFERENCE	−5.6	−4.6	−4.2	−2.8	−0.3	0.5	2.7
NB^(^′^)^ - NT^(^′^)^ CLEFT IMPRESSION 1	1.8	2.5	2.8	2.8	3.0	4.1	0.8
NB^(^′^)^ - NT^(^′^)^ CLEFT IMPRESSION 2	3.7	3.9	4.8	4.9	6.0	6.0	1.1
NB^(^′^)^ - NT^(^′^)^ CLEFT DIFFERENCE	0.7	0.8	1.4	2.1	3.4	4.2	1.6
NB^(^′^)^ - NT^(^′^)^ NON-CLEFT IMPRESSION 1	4.7	5.7	5.8	5.7	5.9	6.3	0.5
NB^(^′^)^ - NT^(^′^)^ NON-CLEFT IMPRESSION 2	5.8	6.1	6.1	6.2	6.5	6.6	0.3
NB^(^′^)^ - NT^(^′^)^ NON-CLEFT DIFFERENCE	0.2	0.3	0.4	0.6	0.6	1.4	0.5
SN - CA IMPRESSION 1	2.3	2.5	3.1	3.5	4.4	5.1	1.2
SN - CA IMPRESSION 2	2.8	3.1	4.0	4.3	5.4	6.3	1.5
SN – CA DIFFERENCE	0.5	0.5	0.6	1.1	1.2	2.7	1.0
SN-CA ∢ AB-AB IMPRESSION 1 [ANGLE]	18.6	25.1	31.4	40.7	46.9	87.8	25.7
SN-CA ∢ AB-AB IMPRESSION 2 [ANGLE]	34.7	43.5	46.2	53.9	64.9	80.3	18.4
SN-CA ∢ AB-AB DIFFERENCE [ANGLE]	−7.6	5.5	13.5	11.8	22.4	24.9	13.3
NR^(^′^)^ - NL^(^′^)^ CLEFT IMPRESSION 1	11.8	14.2	14.9	15.1	16.2	18.2	2.2
NR^(^′^)^ - NL^(^′^)^ CLEFT IMPRESSION 2	10.8	13.3	14.2	13.9	14.3	16.7	2.1
NR^(^′^)^ - NL^(^′^)^ CLEFT DIFFERENCE	−2.3	−1.0	0.1	−0.6	0.1	0.3	1.1
NR^(^′^)^ - NL^(^′^)^ NON-CLEFT IMPRESSION 1	8.0	8.3	8.6	8.8	8.8	10.6	0.9
NR^(^′^)^ - NL^(^′^)^ NON-CLEFT IMPRESSION 2	7.6	8.1	9.4	9.2	9.7	11.1	1.4
NR^(^′^)^ - NL^(^′^)^ NON-CLEFT DIFFERENCE	−1.1	0.1	0.4	0.3	0.6	1.4	0.9

## Discussion

The long-term results with regard to nasal aesthetics and functional improvements are the subject of ongoing discussions^11^. Studies have nevertheless shown that NAM can help to improve nasal symmetry and reduce the extent of follow-up surgeries^21,22^. This investigation introduces a further facilitation of nasal attachment and compares two nasal stent designs. One is used in conventional CAD/CAM-intraoral molding plates, whereas the second one is part of the semi-automated RapidNAM-plate design solution. Despite the different plate and retention-pin designs, the basic techniques used are a derivative of traditional NAM with regular activation of the wire. The use of a wire allows a precise positioning of the nasal bulb^5,8^. The results of this study show that both approaches significantly elevate the nostril on the cleft side with an increase in height of more than 60% and 75%. This is a much greater increase than on the non-affected nostril on the other side. Furthermore, the columella angle was noticeably raised in both groups, whereas the initial values differed between the two groups. The preceding CAD/CAM-cohort started out with a mean of 18.3° that changed to 47.3°. The initial angle of the RapidNAM-group was higher with a mean of 40.7° at the beginning and 53.9° at the end of treatment. The columella was lengthened in the CAD/CAM-NAM group by 55% and in the RapidNAM-group by 31%. However, patients of the RapidNAM had a greater range in the values mentioned above and varied more between each other than did patients from the CAD/CAM-NAM group who showed more homogeneous initial values. However, the overall results and trends were comparable with the outcomes of other studies. This indicates that the aims of nasoalveolar molding, namely the improvement of nasal symmetry, columella elongation, increasing of nostril height on the cleft side and raising the columella angle, have all been achieved^23–25^. The additional correction of nasal symmetry will be increased again when surgical lip closure is performed. This happens because of the reduction of the cleft-size, whereas an overcorrection is recommended by some authors to compensate a partial relapse^26^.

When choosing to use CAD/CAM-technology in NAM therapy, the advantage of the new pin and nasal stent retention design is the easy exchangeability when a new plate is required. The presented solution overcomes previous CAD/CAM-approaches that needed manual stent attachments^27^. Since all RapidNAM-plates have a pin with the same dimensions, a previously fitting nasal stent can be removed by unwinding the screw and can be transferred onto the next plate. The orientation towards the cleft-side nostril will still fit. Prior to overall clinical use of the stent, the length of wire chosen should not be too short, so that further activations throughout the treatment can be carried out without exchanging the stent itself. Though the new retention system is not universally available, it can be designed virtually by experienced users of CAD/CAM-design software who already use CAD/CAM-intraoral molding plates. Once designed, it can be virtually mounted with a Boolean operation onto intraoral molding plates even when manually creating a virtual sequence of plates. With an onsite plate design, however, the pin position can be selected in view of the clinical situation. 3D photography can help additionally to capture the soft-tissue relationships, especially during different facial movements for a suitable pin positon^28^, when the plate has to be designed at a later time.

Further efforts will be directed to a set of nasal stents with various sizes and loop angles that are pre-fabricated. Depending on the clinical situation, the correct size can be picked, and the detailed adjustment can be performed even more quickly. Furthermore, the retentive 180°-knee will be refined to avoid future repetitive reinforcements with resin pattern.

The clinical pilot study of this new nasal stent design will promote efficient and time-saving NAM treatment using CAD/CAM-technology, while still focusing on satisfying clinical results. This is ensured by implementing traditional features of well-established and long-term exercised NAM techniques.

## Conclusion

The introduced quick-lock system for CAD/CAM-NAM devices is the combination of traditional NAM with additive manufacturing. The integration of the pin is part of the RapidNAM algorithm and is very time-efficient. The exchange of the previously fitted nasal stent to another plate is fast and only needs minor corrections thus reducing treatment hours.

### Ethical Statement and Patient Recruitment

All clinical investigations and procedures were conducted according to the principles expressed in the Declaration of Helsinki. Ethical approval for the prospective application study was granted by the Ethical Committee of the Technische Universität München (Approval No. 67/15 S). All interactions with each patient were performed with written parental consent.
